# Density-dependent lineage instability of MDA-MB-435 breast cancer cells

**DOI:** 10.3892/ol.2013.1157

**Published:** 2013-01-28

**Authors:** ANDREAS G. NERLICH, BEATRICE E. BACHMEIER

**Affiliations:** 1 Department of Pathology, Academic Hospital Munich-Bogenhausen, Munich, Germany; 2 Department of Clinical Chemistry and Clinical Biochemistry, Ludwig-Maximilians-University, Munich, Germany

**Keywords:** *in vitro* study, breast cancer cell lines, melanocytic phenotype

## Abstract

The use of cell lines in cancer research is strongly dependent on the avoidance of contaminations, the correct attribution of a cell line to the initial primary tumor and stability. Previous studies have identified expression of melanocytic molecular markers in the widely used breast cancer cell line, MDA-MB-435. In the present study the three breast cancer cell lines, MCF-7, MDA-MB-231 and MDA-MB-435, were systematically analyzed for mRNA and protein expression of major epithelial (cytokeratin isoforms), mammary (mammaglobin) and melanocytic (melan A and S100-protein) markers. Protein expression was identified by immunocytochemistry and quantitative RT-PCR was used to determine mRNA levels. While MCF-7 and MDA-MB-231 cells unambiguously revealed an epithelial/mammary phenotype, MDA-MB-435 cells were found to exhibit epithelial/mammary and melanocytic features dependent on cell density. Subconfluent cells demonstrated epithelial characteristics only, however, densely growing, confluent cells also expressed melanocytic markers. Consistent with gain of melanocytic features, the expression levels of mammaglobin mRNA decreased in these cells. These results indicate that the three cell lines are primarily of epithelial phenotype, however, MDA-MB-435 cells revealed lineage infidelity in dense cultures with a gain in melanocytic phenotype. These characteristics must be taken into consideration when analyzing cancer-relevant genes and their expression profiles *in vitro*.

## Introduction

The use of *in vitro* cell lines is a widely established approach to study the growth properties and regulatory pathways of malignant tumors that are considered important for tumor expansion and spread. This is extremely important in breast cancer studies as a number of well-established cell lines have been in use for several years.

Previously, the application of microarray analyses has enabled clustering of several tumor types into specific groups. These studies did not provide a ‘breast cancer specific’ cluster and the MDA-MB-435 breast cancer cell line was out of range of other breast cancer cell lines, appearing in the same cluster as melanoma cell lines due to expression of specific melanocytic genes (1). Further studies using protein expression analyses clearly demonstrated that two MDA-MB-435 sublines (termed MDA-MB-435S and -HGF) expressed the melanocytic genes melan A and tyrosinase (2), but not typical breast cancer genes, mammaglobin, prolactin and pS2. Rae *et al*(3) revealed that a number of MDA-MB-435 cell lineages used in various laboratories were genetically identical to the parental cell line and also exhibited a melanocytic phenotype. Collectively, these studies reported that the widely used breast cancer model cell line MDA-MB-435 does not reflect a breast carcinoma cell line and may originate from a metastasis from an undetected malignant melanoma.

By contrast, other studies have reported clear evidence that MDA-MB-435 cells produced milk lipid droplets following differentiation (4). In addition, the cell line has been found to express epithelial markers, including pan-cytokeratin (CK), indicative of an epithelial origin (5). Finally, an extensive study by Sellappan *et al*(6) confirmed the epithelial characteristics of those cells based on the production of typical milk lipid droplets together with the expression of pan-cytokeratin, epithelial membrane-antigen (EMA), β-casein and α-lactalbumin. However, this cell line also revealed specific expression of the melanoma markers tyrosinase and melan A. The authors hypothesized that the MDA-MB-435 cells exhibited lineage instability and classified them as a breast epithelial cell line that had undergone lineage infidelity (6).

These observations are consistent with our own previous study (7) in which 159 breast cancer samples were analyzed for melanocyte and epithelial markers and CKs. These tumors were identified to contain a small subpopulation of cells with high expression levels of Melan A. However, the immunohistochemical identification of focal positivity for Melan A in otherwise (clinically) clear-cut breast carcinoma must not be interpreted as melanoma metastases. The association between Melan A expression and differentiation grading indicates that lineage infidelity correlates with a reduction in cellular differentiation and therefore, Melan A may be an important marker for reduction in tumor cell differentiation (7)

The current study is a comparative study of MDA-MB-435 (directly obtained from the initial, unmodified cell line) and additional commonly used breast cancer cell lines (MCF-7 and MDA-MB-231), aiming to extend our previous studies. The expression of breast cancer and/or melanocytic marker profiles in various growth stages of breast cancer cells was determined by quantification of gene and protein expression techniques. Results demonstrate that lineage infidelity of MDA-MB-435 cells is associated with tumor cell density, indicating that the growth properties of these cells have an effect on the gain of melanocytic properties. The observation that gene and protein expression levels change with cell density has been previously reported by this research group. In these studies, breast cancer cells growing in cultures with low density were demonstrated to exhibit a more aggressive phenotype with elevated proteolytic activity and invasiveness (8–10).

In the present study, MDA-MB-435 cells growing in dense cultures were found to exhibit lower expression of the breast cancer marker, mammaglobin and higher expression of the melanocyte markers, Melan A and S100.

The results are consistent with the hypothesis that MDA-MB-435 cells were initially of epithelial origin and therefore suitable for the study of breast cancer. Furthermore, for the first time, cell density was demonstrated to correlate with differentiation state in breast cancer and additional evidence that Melan A is a suitable marker for differentiation in breast cancer is provided.

## Materials and methods

### Cell culture conditions

The breast cancer cell lines MCF-7, MDA-MB-231 (both American Type Culture Collection, Manassas, VA, USA) and MDA-MB-435 (kindly donated by Dr Janet E. Price, Anderson Cancer Center, Houston, TX, USA) used in this study reflect a stepwise increase in malignant biological behavior on the basis of methodical retransplantation studies, MDA-MB-435>MDA-MB-231>MCF-7 (11,12). These cell lines are commonly used for breast cancer studies and therefore are well-defined in their growth, invasive and metastatic characteristics.

Cells were cultured at 37°C in a humidified atmosphere of 5% CO_2_ and 95% air. For comparative analysis of melan A mRNA expression, the established melanoma cell line A2058 was also included (American Type Culture Collection). Cells were grown in MEM (Eagle’s) with Earle’s salts supplemented with 20% heat inactivated fetal calf serum, 1% L-glutamine solution (200 mM), 1% sodium pyruvate solution (100 mM), 1% non-essential amino acids for MEM (100X solution) and 2% vitamins for MEM (100X solution). The medium was changed every three days. For subcultures, cells were harvested following brief treatment with 0.1% trypsin/EDTA solution and seeded at a dilution of 1:5. Cells of 10 subsequent passages were used for studies.

Cells were grown to subconfluency (<50% of confluence) or ∼90% confluence in 75-cm^2^ plastic culture flasks containing 15 ml medium.

### Immunocytochemistry

Immunocytochemistry was used to determine the cellular pattern of pan-CK (clone Lu-5) and various CK isoforms (KL-1 and CK-10, -14, -18, -19 and 20), the breast cancer marker, mammaglobin and the melanocytic markers, S100 and melan-A. Antibodies against the CKs were all purchased from Dako (Hamburg, Germany). Monoclonal antibodies against melan A, mammaglobin and S100 were obtained from Zytomed (Berlin, Germany).

Cells were grown to subconfluency or confluency on silanized sterile glass slides (SuperFrost plus; Menzel, Braunschweig, Germany). The medium was discarded, the cells rinsed with Tris-buffer, fixed in methanol/acetone (2:1 v/v) for 2 min and rinsed again. Following incubation with the specific polyclonal primary antibodies for 30 min (37°C), rinsing with Tris-buffer and application of the secondary antibody system (Streptavidin-Biotin-Complex method; Dako), the resulting antibody complexes were visualized with diaminobenzidine (Sigma-Aldrich, Deisenhofen, Germany).

### Quantitative RT-PCR

RNA was extracted from cells using the RNeasy Protect Mini kit (Qiagen, Hilden, Germany) according to the manufacturer’s instructions and reverse transcribed using oligo dT primers in 20 *μ*l final volume. All primers for the genes tested were designed using Primer3 software (13) with a T_m_ optimum of ∼60°C and a product length of 100–150 nt (Table I). Real time PCR was performed on an I-Cycler (Bio-Rad, Hercules, CA, USA) using iQ Supermix (Bio-Rad) supplemented with 10 nM fluorescein (Bio-Rad), 0.1X Sybr-Green I (Sigma-Aldrich), 2.5 *μ*l cDNA (5X diluted) and 3 pmol sense and antisense primers in a final reaction volume of 25 *μ*l. Following an initial denaturation step of 3 min during which the well factor was measured, 50 cycles of 15 sec at 95°C followed by 30 sec at 60°C were performed. Fluorescence was measured during the annealing step in each cycle. Following amplification, melting curves with 80 steps of 15 sec at 0.5°C increments were performed to monitor amplicon identity. Amplification efficiency was assessed for all primer sets utilized in separate reactions and primers with efficiencies >94% were used. Expression data were normalized against GAPDH and on RNA polymerase II (RPII) gene expression data obtained in parallel. Results are expressed with standard errors and statistical comparisons (unpaired two-tailed t-test) were obtained using Qgene software (14). Expression changes were calculated using the mean value of normalizations obtained using GAPDH and RPII genes as references.

### Statistical analysis

Statistical significance was assessed by comparing mean ± SD values, which were normalized to the control group with student’s t-test for independent groups. One-way analysis of variance was used to test for statistical significance and when significance was determined, Bonferroni’s multiple comparison test was performed post hoc. P<0.05 was considered to indicate a statistically significant difference. Statistical analysis was performed using the Prism software (GraphPad, San Diego, CA, USA).

## Results

### Expression pattern of CKs in breast cancer cell lines of various levels of tumorigenicity and growth density

Expression of CKs were analyzed to verify the epithelial origin of the three human breast cancer cell lines, MCF-7, MDA-MB-231 and MDA-MB-435, which constitute our *in vitro* cell model. All cell lines have been previously reported to originate from pleural effusions (12,15). MCF-7 cells have been found to exhibit low invasive abilities and are essentially non-metastatic. By contrast, injection of MDA-MB-231 and -435 cells into the mammary fat pad of nude mice has been demonstrated to result in tumor formation and distant metastases in lungs and lymph nodes of specific mice (12), however, the extent of these events varied between cell lines and growth velocity differed.

**Figure 1:**
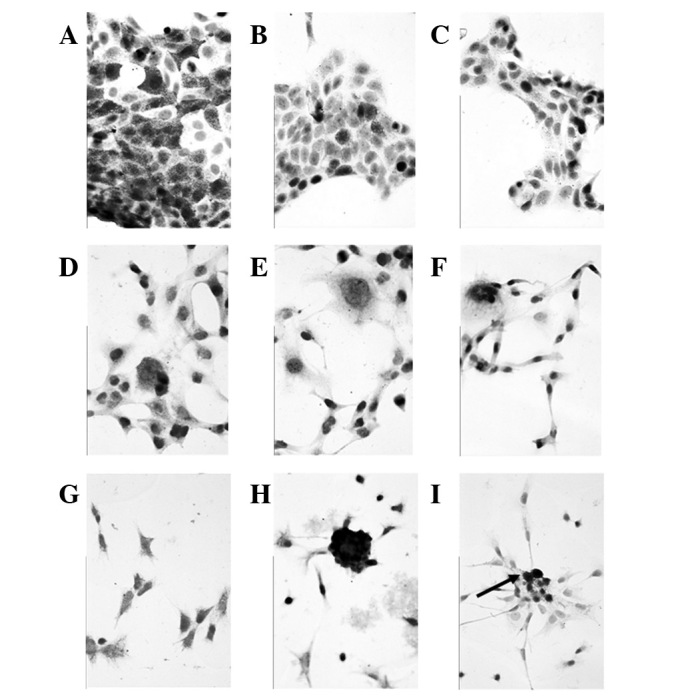
Immunocytochemical staining pattern for cell-associated epithelial and melanocytic markers in (A–C) MCF-7, (D–F) MDA-MB-231 and (G–I) MDA-MB-435 cells. Cytoplasmic staining against (A,D,G) pan-CK, (B,E,H) mammaglobin and (C,F,I) melan A. MCF-7 and MDA-MB-231 cells were negative for melan 1, while MDA-MB-435 cells reveal a focal typical positive reaction for this melanocytic marker, particularly in regions with accumulated, ‘denser’ tumor cells (magnification, ×400). CK, cytokeratin.

All three human breast cancer cell lines exhibited the epithelial marker, pan-CK (clone Lu-5) and the low-molecular weight CK, KL-1 (Fig. 1). However, there were line-specific differences in the tested CK isoforms. Similarly, MCF-7 revealed specific positive staining for CK-18, -19 and -20 and the MDA-MB-231 cells for CK-18 and -19. The MDA-MB-435 cells, however, did not react for CK18, -19 or -20. All three cell lines were negative for CK-10 and -14 (squamous cell differentiation markers; Table II). These patterns were identified in the subconfluent and confluent growth conditions investigated in the three cell lines.

### Expression pattern of mammaglobin in the various cell lines

Immunohistochemistry was used to analyze mammaglobin protein expression in the cell lines (Fig. 1). As identified for pan-CK, all cell lines were positively stained (Table II). No differences in expression were found between subconfluent and confluent cells.

### Expression of melanocytic expression markers in the various cell lines

While CKs and mammaglobin protein were found in all three cell lines, the melanocytic markers melan A and S100-protein were negative in MCF-7 and MDA-MB-231 cells of subconfluent and confluent growth status. However, MDA-MB-435 cells were negative for these markers under subconfluent conditions only, while in confluent cell cultures a focal specific cytoplasmic staining for melan A and S100 was detected. Staining was observed to be higher in areas where densely grown cells formed cluster-like aggregates (Table III and Fig. 1).

### Quantitative mRNA expression of mammaglobin in the cell lines

**Figure 2:**
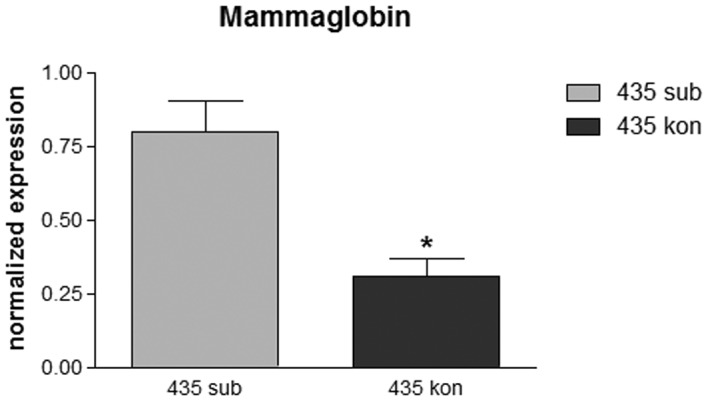
mRNA expression levels for mammaglobin in MDA-MB-435 cells of various tumor cell densities. Transcription rate in the sub cells was high and a significantly lower level was observed in the kon cultures. Differences in expression levels were statistically significant (^*^P<0.05, vs. 435 sub; student’s t-test). sub, subconfluent; kon, dense confluent.

To verify the immunohistochemical protein pattern, quantitative RT-PCR was performed to determine mRNA expression of mammaglobin in various settings. While MCF-7 and MDA-MB-231 cells revealed moderate expression of mammaglobin at various levels of confluence without significant differences (data not shown), MDA-MB-435 cells revealed statistically significant differences between cell densities. The mRNA level for mammaglobin was ∼3-fold higher in subconfluent than in confluent cells (Fig. 2).

### Quantitative mRNA expression of melan A in various breast cancer cell lines

**Figure 3:**
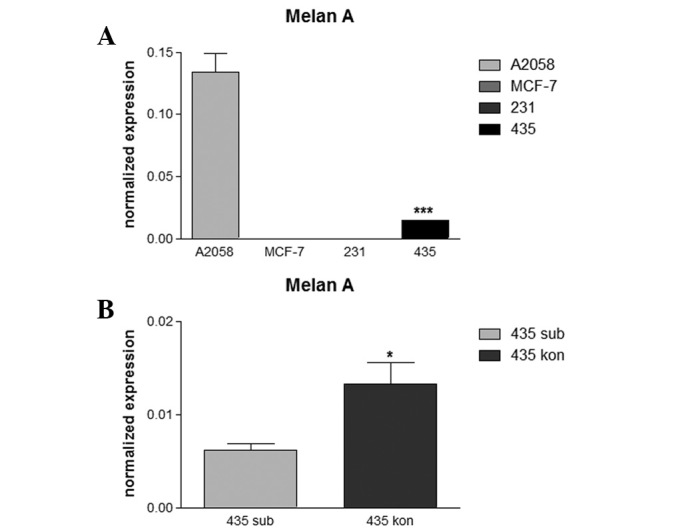
(A) mRNA expression levels for melan A in various breast cancer cell lines of confluent tumor cell density, revealing strong expression of melan A in the melanocytic cell line, A2058 and minor expression in MDA-MB-435 cells. MCF-7 and MDA-MB-231 cells did not express melan A. Differences in expression levels were significant (^***^P<0.001, cell line MDA-MB-435 vs. MDA-MB-231 and MCF-7; analysis of variance with Bonferroni’s post test). (B) mRNA expression levels for melan A in MDA-MB-435 cells of various cell densities. Significant increase in melan A in kon compared with sub cells. Differences were statistically significant (^*^P<0.05, vs. 435 sub; student’s t-test). sub, subconfluent; kon, dense confluent.

Analysis of melan A mRNA expression levels revealed an absence of this melanocytic marker in MCF-7 and MDA-MB-231 cells (Fig. 3A), consistent with immunohistochemistry results. In MDA-MB-435 cells, a low level of melan A expression was observed in subconfluent cell cultures which was found to be significantly increased ∼2-fold in the dense cultures (Fig. 3B). The melanocytic cell line A2058 was used as control and revealed high expression levels of melan A which exceeded levels in confluent MDA-MB-435 cells by >10-fold (Fig. 3A). Expression of melan A mRNA was not detected in tthe MCF-7 and MDA-MB-231 cell lines.

## Discussion

*In vitro* cell lines as model systems are an extremely broad application for cancer research. However, the results obtained from using cell lines are markedly dependent on prevention of contamination, correct attribution of a cell line to the initial primary tumor and stability. The correct identification of the breast cancer cell line, MDA MB-435, a widely used cell line in breast cancer research remains an area of fierce debate. Similar to several other breast cancer cell lines, MDA MB-435 cells originate from the pleural effusion of a patient who succumbed to mammary carcinoma (11,16).Establishing a permanent cell line from a primary tumor is extremely difficult, while metastatic tumor cells, particularly those that have detached from the surrounding stroma and are freely floating in effusion fluids, are significantly easier to handle. However, in rare instances, the metastases may not occur from the predicted primary tumor and may originate from an unknown synchronous malignant tumor that systemically spreads more readily.

The development of molecular array techniques has enabled high throughput analysis of thousands of genes and a number of tumors and tumor cell lines have been molecularly screened to analyze gene expression pattern. Ross *et al*(1) performed an extensive study on the expression levels of various tumor cell lines. By this approach, MDA-MB-435 cells were identified to express typical markers of melanocytic origin. As a consequence, those authors hypothesized that the MDA-MB-435 cells may have originated from an unknown metastasizing malignant melanoma which occurred in parallel to a (non-metastazing) breast cancer. In addition, two subsequent studies on *in vitro* cells and tumor tissue obtained following tumor cell transplantation into nude mice (2,3), exclusively detected melanocytic markers in MDA-MB-435 cells and transplanted tumor nodules.

However, other studies performed morphology analysis and immunocytochemistry and demonstrated that MDA-MB-435 cells expressed epithelial cell markers, in particular milk lipid droplets (4) and CKs (5). In addition, a previous study by Sellappan *et al*(6) identified epithelial cell markers (CKs) and breast epithelia-specific genes, including β-casein and α-lactalbumin, in MDA MB-435 cells. In this study, authors observed expression of melanocytic markers, tyrosinase and melan A, in those cells, hypothesizing that lineage infidelity had occured during tumor progression.

The results of the present study are consistent with observations of Sellappan *et al*, demonstrating that MDA-MB-435 cells coexpress breast epithelia-specific (CKs and mammaglobin) and melanocytic (melan A and S100) markers. In addition, the locally invasive, but non-metastasizing, estrogen receptor-positive MCF-7 cell line and the invasive and moderately metastasizing, estrogen receptor-negative MDA-MB-231 cell line were screened for lineage instability. Neither of the cell lines revealed a melanocytic phenotype.

To characterize the cell lines with respect to their epithelial origin, the expression of various CKs was analyzed to determine the type and degree of cellular differentiation and maturation (17). By this approach, the non-invasive MCF-7 cells were found to be ‘more differentiated’ than the invasive MDA-MB-231 cells, since the MCF-7 cells synthesized various glandular CKs, including CK-20, which was not identified in the MDA-MB-231 cells. The highly aggressive MDA-MB-435 cell line was not found to express glandular or squamous CKs and assumed to exhibit a significantly lower degree of differentiation.

In addition, the expression patterns for epithelial and melanocytic markers were compared in all three breast cancer cell lines with respect to cell density and thereby the growth status of the cells. MCF-7 and MDA-MB-231 cells revealed a stable epithelial phenotype, however, MDA-MB-435 cells reacted differently when subconfluent and confluent cells were compared. Subconfluent cells were observed to have undergone epithelial differentiation, however, in dense cultures, foci which had undergone melanocytic differentiation were identified. This observation is consistent with the tumor transplantation experiments by Sellapan *et al*(6) in which small foci of the tumor transplant were detected to express the melanocytic marker HMB-45. In the present study, mammaglobin mRNA expression was found to decrease, concomitant with a gradual increase of melan A mRNA in fully confluent cells. These observations are consistent with coexpression of the epithelial marker, EMA and the melanocytic marker, HMB-45, reported in a previous study in tumor transplants (6). These observations markedly indicate a dual differentiation status of densely packed cells.

In summary, the present study clearly demonstrates that MDA-MB-435 cells undergo a high level of lineage instability leading to the gain of melanocytic lineage characteristics. This instability is markedly dependent on the growth stage of cells and occurs only in foci of densely growing tumor cells. There is clear evidence that the MDA-MB-435 cells are of epithelial origin and therefore no metastasis of an unknown malignant melanoma has been hypothesized to be included in the initial pleural effusion where the cells were obtained. In addition, lineage instability was not found in the other breast cancer cell lines, MCF-7 and MDA-MB-231, at subconfluent or confluent growth stages. Cell density was observed to correlate with differentiation status in breast cancer and is consistent with the hypothesis that Melan A is a suitable marker for differentiation in breast cancer.

Finally, our observations provide a significant evidence for the further use of MDA-MB-435 cells as breast cancer cells in various model systems, in particular in those that correlate molecular observations with tumor cell behaviour (9). However, additional studies must be performed to identify the molecular mechanism of this lineage instability, including the microdis-section of melanocytic versus epithelial MDA-MB-435 cells to understand the molecular and/or genetic background of this unusual observation.

## Figures and Tables

**Table I:** Primer sequences for mammaglobin and melan A.

Gene	Sense	Antisense
Mammaglobin	5′-TCCAAGACAATCAATCCACAAG-3′	5′-CAGTTCTGTGAGCCAAAGGTC-3′
Melan A	5′-ATCGGGACAGCAAAGTGTCTC-3′	5′-GAGTTTCTCATAAGCAGGTGGAG-3′

**Table II:** Immunocytochemical staining for CKs and mammaglobin in breast cancer cell lines.

	Breast cancer cell lines		
Target	MCF-7	MDA-MB-231	MDA-MB-435
LMW-CK (KL-1)	+	+	+
CK-10	0	0	0
CK-14	0	0	0
CK-18	+	+	0
CK-19	+	+	0
CK-20	+	0	0
Mammaglobin	+	+	+

+, positive in all/majority of cells; 0, absent. CK, cytokeratin; LMW, low molecular weight.

**Table III:** Immunocytochemical staining for melanocytic markers in MDA-MB-435 cells.

	Cell confluency	
Markers	Subconfluent	Densely packed (confluent)
Melan A	0	+
S100-protein	0	+

+, positive in all/majority of cells; 0, absent.
